# Supplementary figures and images for: Korean Validation of the Short Version of the TEMPS-A (Temperament Evaluation of Memphis, Pisa, Paris, and San Diego Autoquestionnaire) in Patients with Mood Disorders
Source: Medicina (Kaunas). 2023 Jan 6;59(1):115. doi: 10.3390/medicina59010115 (PMC9860563; doi:10.3390/medicina59010115)

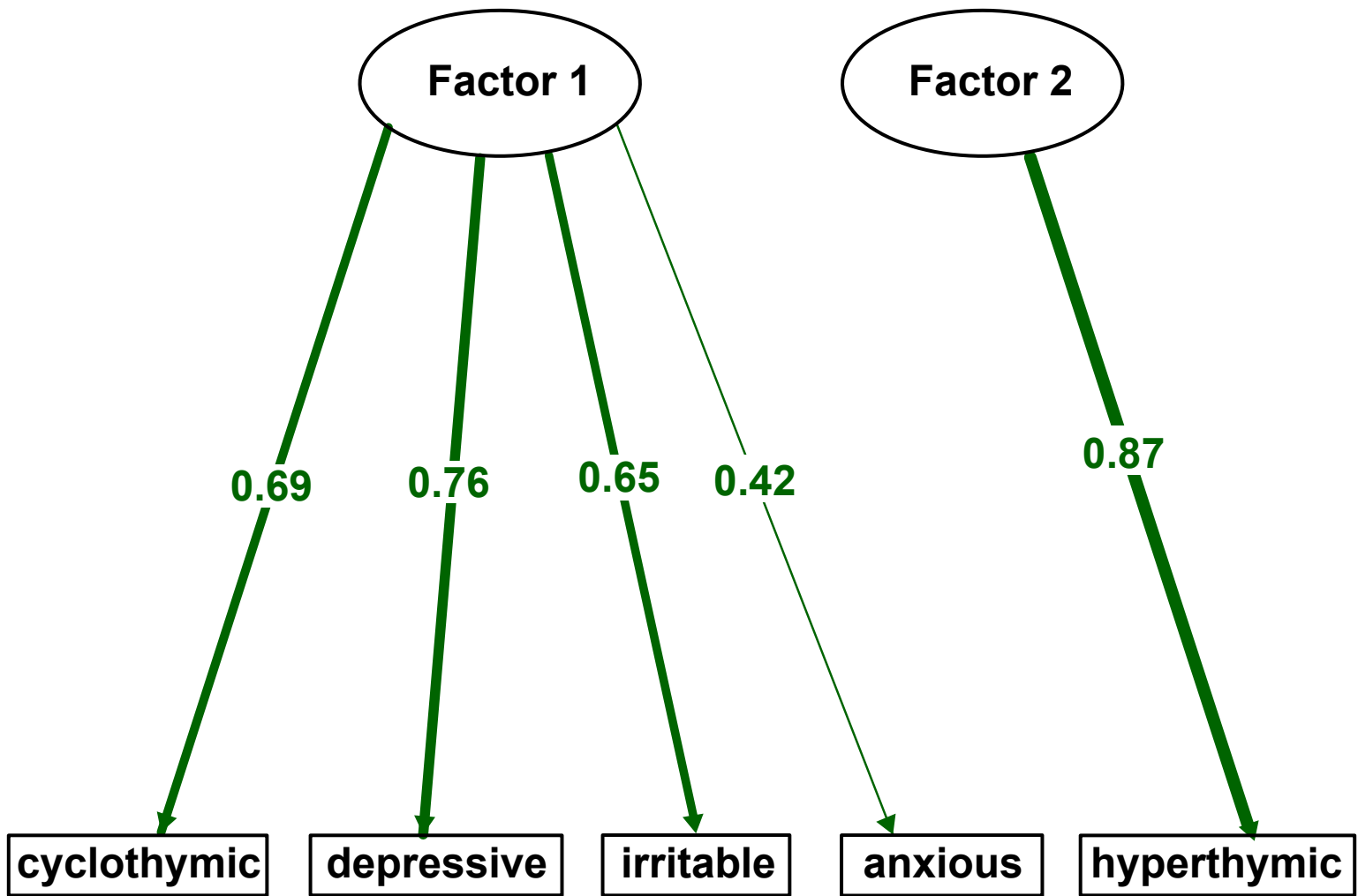

Supplement: Supplementary file 1 [file medicina-59-00115-s001.zip › medicina-2109501-supplementary.pdf]
